# The miRNAome of globe artichoke: conserved and novel micro RNAs and target analysis

**DOI:** 10.1186/1471-2164-13-41

**Published:** 2012-01-24

**Authors:** Domenico De Paola, Federica Cattonaro, Domenico Pignone, Gabriella Sonnante

**Affiliations:** 1 Institute of Plant Genetics (IGV), National Research Council (CNR), Via Amendola 165/A, 70126 Bari - Italy; 2 Istituto di Genomica Applicata (IGA), Via Linussio 51, 33100 Udine - Italy

## Abstract

**Background:**

Plant microRNAs (miRNAs) are involved in post-transcriptional regulatory mechanisms of several processes, including the response to biotic and abiotic stress, often contributing to the adaptive response of the plant to adverse conditions. In addition to conserved miRNAs, found in a wide range of plant species a number of novel species-specific miRNAs, displaying lower levels of expression can be found. Due to low abundance, non conserved miRNAs are difficult to identify and isolate using conventional approaches. Conversely, deep-sequencing of small RNA (sRNA) libraries can detect even poorly expressed miRNAs.

No miRNAs from globe artichoke have been described to date. We analyzed the miRNAome from artichoke by deep sequencing four sRNA libraries obtained from NaCl stressed and control leaves and roots.

**Results:**

Conserved and novel miRNAs were discovered using accepted criteria. The expression level of selected miRNAs was monitored by quantitative real-time PCR. Targets were predicted and validated for their cleavage site. A total of 122 artichoke miRNAs were identified, 98 (25 families) of which were conserved with other plant species, and 24 were novel. Some miRNAs were differentially expressed according to tissue or condition, magnitude of variation after salt stress being more pronounced in roots. Target function was predicted by comparison to *Arabidopsis *proteins; the 43 targets (23 for novel miRNAs) identified included transcription factors and other genes, most of which involved in the response to various stresses. An unusual cleaved transcript was detected for miR393 target, transport inhibitor response 1.

**Conclusions:**

The miRNAome from artichoke, including novel miRNAs, was unveiled, providing useful information on the expression in different organs and conditions. New target genes were identified. We suggest that the generation of secondary short-interfering RNAs from miR393 target can be a general rule in the plant kingdom.

## Background

MicroRNAs (miRNAs) are a class of small RNAs (sRNAs), generally 21-24 nucleotide in length, involved in post-transcriptional regulatory mechanisms of several processes, including the response to biotic and abiotic stress [1,2]. In plants, miRNAs derive from non-coding transcripts produced from MIR genes, mainly located in intergenic regions. The primary transcripts (pri-miRNA) possess internal stem-loop secondary structures, which form parts of double-stranded RNA (dsRNA) not perfectly complementary to miRNA, contained in one of the two arms of the stem-loop structure. The pri-miRNA is processed into a stem-loop precursor (pre-miRNA), which is cut by the enzyme DCL1 into a small dsRNA, composed of the mature miRNA and its complementary sequence (miRNA*). The couple miRNA/miRNA* contains protruding extremities, with two unaligned nucleotides at the 3' end. After incorporation into AGO1 protein complex, plant mature miRNAs target mRNAs, which are cleaved by AGO1 at a specific position, opposite to the 10^th ^and 11^th ^nucleotides of the miRNA [3].

In plants, 25 miRNA families are highly conserved and can be found even in distantly related species. However, there is a number of species-specific miRNAs originating from recently evolved MIR genes [4]. Young miRNAs are usually associated with low expression levels [5,6], and are therefore difficult to detect using conventional methods for miRNA identification.

For the isolation of miRNAs in a plant species, one of the approaches, followed since the beginning in *Arabidopsis *and rice, is based on cloning and sequencing small RNA fractions [7,8]. Once miRNA sequences have accumulated in the public databases, computational strategies have been developed to identify miRNAs by sequence comparison. Conserved miRNA sequences from miRBase (http://www.mirbase.org/) are blasted against available genomic/mRNA sequences and searched for sequence similarity [9,10]. Both these approaches are able to detect the most abundant miRNAs belonging to the most ancient and conserved families [11]. More recently, the preferred strategy for the discovery of miRNAs has been based on deep sequencing; in this case, even poorly expressed miRNAs can be detected, therefore allowing the discovery of novel species-specific miRNAs [6,12-17].

It is well known that plants cope with saline stress by activating a number of genes involved in a broad spectrum of metabolisms [18], and miRNAs are involved in the response to environmental stresses [19]. Most studies have been conducted in *Arabidopsis*, where the expression of several miRNAs has been associated to drought tolerance [20]. miRNAs involved in the response to salt treatment have been described, besides *Arabidopsis *[21,22], also in crop species [13,23-29].

Globe artichoke is an important vegetable crop in the Mediterranean region from where it originated [30], and is also widely cultivated in California, Peru and China (http://faostat.fao.org). Artichoke is considered a nutraceutical food since it possesses antioxidant activity attributed to the presence of polyphenols, particularly caffeoylquinic acids and flavonoids [31-34].

Although artichoke can be regarded as a moderately salt resistant crop, the need to maintain low soil salinity levels is essential for maximum yields, since when salt concentration becomes too high, the size of the buds decreases [35]. In the areas where artichoke is more diffused, the increase of saline content in the soils and/or in the water used for irrigation can be a serious problem in a perspective of global climate change. Therefore, understanding which are the mechanisms superintending saline response is of pivotal importance for developing strategies for plant cultivation in future times.

In this study, we deep sequenced the sRNA fraction from leaves and roots of globe artichoke, under standard conditions and under salt stress. Putative miRNA sequences were blasted against artichoke ESTs and Illumina genomic sequences obtained by our group [36]. These analyses led to the identification of 122 (98 conserved and 24 novel) artichoke miRNAs, following stringent criteria. In some cases, MIR genes were detected and miRNA folding structure predicted. Some miRNAs were experimentally validated and their differential expression level was assessed by means of quantitative real-time PCR (qPCR). Target prediction was performed and, when possible, target cleavage was experimentally validated.

## Results

### Artichoke sRNA population

**Figure 1:**
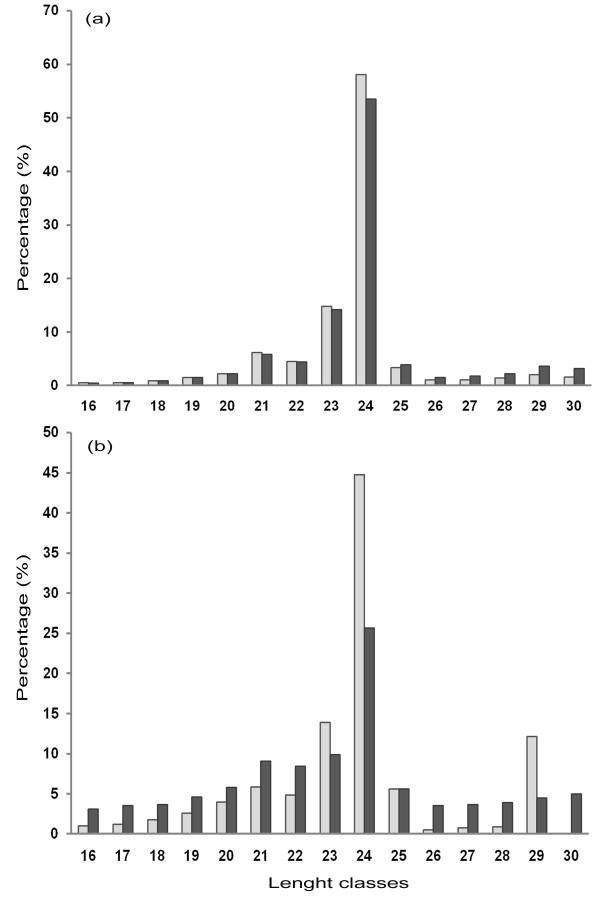
**Small RNA read size**. Distribution and abundance of small RNA unique sequences in leaves (a) and roots (b). Light bars indicate control plants; dark bars refer to NaCl stressed plants.

To discover the artichoke miRNAome and the possible involvement of miRNAs in the response to saline stress, four sRNA libraries were generated from leaves and roots of young artichoke plants subjected or not to NaCl treatment. Deep sequencing yielded a total of almost 28 million reads (Table 1). After removing low quality reads, adapter sequences, and sequences shorter than 16 or longer than 30 nucleotides, reads were about 6 million (corresponding to 2,447,091 unique sequences) for control plant (CP) leaves, almost 7 million (2,151,783 unique sequences) for stressed plant (SP) leaves, almost 3 million (1,194,486 unique sequences) for CP roots, and about 3 million (734,396 unique sequences) for SP roots (Table 1). The length distribution of unique reads revealed a non homogeneous pattern, the majority of sRNAs in all libraries (on average 79%) being 20-25 nucleotide in length with 24-nt long sRNAs as the main peak (Figure 1). Reads from all artichoke libraries were annotated according to small non-coding RNAs contained in Rfam (Additional File 1). As a result, 16.16%, 19.88%, 20.32%, and 44.72% reads matching known sRNAs were identified in CP leaves, SP leaves, CP roots and SP roots, respectively. The most abundant class was rRNA, in agreement with the method used to obtain sRNA fraction for library preparation. tRNAs ranged from a minimum of 2.34% in CP roots to a maximum of 5.55% in SP roots; snRNA and snoRNA seemed to be equally distributed in the four sRNA libraries.

**Table 1:** Sequencing results of sRNA libraries from artichoke

Library	Read counts	Read counts (16-30 nt)	Unique read counts (16-30 nt)
Leaf	7,175,250	5,991,907	2,447,091

Stressed leaf	9,313,867	6,780,853	2,151,783

Root	3,110,135	2,900,558	1,194,486

Stressed root	7,979,595	2,979,640	734,396

nt: nucleotides

### Discovery of artichoke conserved and novel miRNAs

To identify artichoke miRNAs conserved also in other plant species, all previously known plant sequences were extracted from the miRNA Registry Database (miRBase Release 17.0, April 2011), accounting for a total of 3,362 annotated miRNAs from 46 plant species. As a result, 98 known miRNAs (91 and 93 in CP and SP leaves respectively, 77 and 62 in CP and SP roots respectively) were identified in artichoke, with 54 of them being shared by all four sRNA libraries (Additional File 2). The artichoke miRNAs were named using the initials of the species (cca for *Cynara cardunculus*) followed by the number (and letter when applicable) assigned to the same miRNA in other plant species.

The 98 known artichoke miRNAs belonged to 25 families, of which 20 had at least two members, while 5 had only one member (Additional File 2). Highly represented miRNA families showed one or more dominant members with high read counts (e.g. cca-miR156a and cca-miR156b) and less represented members having few read counts (e.g. cca-miR156a*; Additional File 2).

Conserved miRNAs identified by deep sequencing were used for BLASTn search against public artichoke ESTs and our proprietary Illumina genomic sequences, and the matching sequences were subjected to Mfold analysis for fold-back structure prediction. After these analyses, 25 pre-miRNAs were identified, corresponding to 19 different miRNA families (Table 2, Additional File 3). Mature miRNAs were either located in the 5' or in the 3' arm of the pre-miRNA sequences (Additional File 3). In three cases, namely cca-miR160, cca-miR169a, and cca-miR395b, two distinct sequences corresponding to putative precursors for each miRNA were found, therefore, following instructions in miRBase, we considered them as distinct miRNAs: cca-miRNA160-1 and 160-2; cca-miRNA169a-1 and 169a-2; cca-miR395b-1 and cca-miR395b-2.

**Table 2:** Precursors of conserved and novel miRNAs from globe artichoke

miRNA	Sequence	pre-miRNA length	MFEI	Accession
cca-miR156a	UGACAGAAGAGAGUGAGCAC	102	1.29	JN381965

cca-miR156a*	GCUCACUGCUCUAUCUGUCACC			

cca-miR156b	UGACAGAAGAGAGUGAGCACA	118	0.88	GE609552

cca-miR157a	UUGACAGAAGAUAGAGAGCAC	98	1.20	JN381966

cca-miR160-1	UGCCUGGCUCCCUGUAUGCCA	96	1.13	JN381967

cca-miR160-2	UGCCUGGCUCCCUGUAUGCCA	93	1.00	JN381968

cca-miR164a	UGGAGAAGCAGGGUACGUGCA	83	0.99	JN381969

cca-miR166d	GGAAUGUUGUCUGGCUCGAGG	86	1.12	JN381970

cca-miR166d*	UCGGACCAGGCUUCAUUCCUU			

cca-miR167a	UGAAGCUGCCAGCAUGAUCUGG	218	0.77	GE597437

cca-miR168a	UCGCUUGGUGCAGGUCGGGAA	118	0.83	JN381971

cca-miR169a-1	CAGCCAAGGAUGACUUGCCGG	102	0.94	JN381972

cca-miR169a-2	CAGCCAAGGAUGACUUGCCGG	99	0.91	JN381973

cca-miR171a	UGAUUGAGCCGUGCCAAUAUC	92	0.82	JN381974

cca-miR172a	AGAAUCUUGAUGAUGCUGCAU	79	1.04	JN381975

cca-miR319c	UUGGACUGAAGGGAGCUCCCU	198	1.11	JN381976

cca-miR390	AAGCUCAGGAGGGAUAGCGCC	88	1.39	JN381977

cca-miR393a	UCCAAAGGGAUCGCAUUGAUCC	142	0.97	JN381978

cca-miR394	UUGGCAUUCUGUCCACCUCC	211	0.81	GE603351

cca-miR395a	CUGAAGUGUUUGGGGGAACUC	80	0.97	JN381980

cca-miR395b-1	CUGAAGUGUUUGGAGGAACUC	92	1.17	JQ029164

cca-miR395b-2	CUGAAGUGUUUGGAGGAACUC	92	1.04	JQ029165

cca-miR396a	UUCCACAGCUUUCUUGAACUU	119	1.38	JN381981

cca-miR396a*	GUUCAAUAAAGCUGUGGGAAA			

cca-miR396b	UUCCACAGCUUUCUUGAACUG	110	1.04	JN381982

cca-miR398a	UGUGUUCUCAGGUCGCCCCUG	100	1.15	GE610628

cca-miR408a	UGCACUGCCUCUUCCCUGGCU	107	0.70	GE605886

cca-miR399a	UGCCAAAGGAGAUUUGCCCUG	83	1.12	JN381983

cca-novel-1-5p	UGUCUAAGACAACUCCUUGGA	117	1.17	JN381984

cca-novel-1-3p	CAAGAAGUUGUCUUAGGCAUG			

cca-novel-2	AUACGACAAAUAGAACAAAUAAAC	71	0.80	JN381985

cca-novel-3	ACGAAAACAUGUUGGUCUCACGUG	217	0.78	JN381986

cca-novel-4-5p	UUGCAAGUAUCCGGAUUUAAA	210	0.78	JN381987

cca-novel-4-3p	UUAAAUCCGGAUACUUGCAAC			

cca-novel-5	AAAGGGGACAAUAUCUGGUACGGU	110	1.38	JN381988

cca-novel-6	CACGAAAACAGACUGGUCUCACA	222	0.94	JN381989

cca-novel-7-1	UGAGAAGCGUAAGAAGGGAUC	157	0.88	JN381990

cca-novel-7-2	UGAGAAGCGUAAGAAGGGAUC	98	0.92	JN381991

cca-novel-7-3	UGAGAAGCGUAAGAAGGGAUC	171	0.72	JN381992

cca-novel-8	AUGGACGUGUUAUUCAUCAUGAAU	122	1.15	JN381993

cca-novel-9-5p	AUCUUGUAACAUUUGAUGAUGUGG	122	1.19	JN381994

cca-novel-9-3p	AUCCACGUCAUCAAAUGUUACAAG			

cca-novel-10-5p	UCUUUAUGUCACGAUGUAUGAC	255	0.86	JN381995

cca-novel-10-3p	CAUGCAUGGUGAUAUAAAUAGC			

cca-novel-11	GAAGUUUCAAGUGUAAAAAAGUGG	116	1.58	JN381996

cca-novel-12	UCUGAAACUCAAGAACACGUUG	81	1.06	JN381997

cca-novel-13-5p	UGAAAGGAAUCAUGAACGUGA	115	0.83	JN381998

cca-novel-13-3p	UCACGUCCAUUGUCCCUUUCA			

cca-novel-14	AAGCGUAAGAAGAGAUCUGAACC	157	0.80	JN381999

cca-novel-15	AUAAGGAGAGUUAAGCUGAGAAGC	184	0.72	JN382000

cca-novel-16-5p	GUAAGAAGAGAUCUCCACCCUUGG	162	0.84	JN382001

cca-novel-16-3p	AUCAAGGGUUCAGAUCUCUUCUU			

cca-novel-17	UAUGGUGAGAAGGGUAAGAAG	169	0.90	JN382002

cca-novel-18	UCUGGACGGUAUGCACAUGUGCAU	140	1.23	JN382003

cca-novel-19	UUCAAGAAAGCUGUGGGAAAA	124	1.34	JN382004

cca-novel-20-5p	CAUGCUUGUGAUCAAAUGAUG	179	0.84	JN382005

cca-novel-20-3p	UCAUUUGAUCACAAGCAUGAG			

cca-novel-21	GGUUAGGUUGAUCGGGUUGAAGAC	98	0.88	GE597304

cca-novel-22-5p	UGGAAUUGGGUGCUUCGGAAGA	116	0.84	GE599895

cca-novel-22-3p	UUCCGAGGCCACCCAUUCCAAC			

MFEI: minimal folding free energy index

Out of the 98 conserved miRNAs identified in artichoke, 46 were in common with several plant species, while 43 were present in only one of the 46 species in miRBase release 17.0. Thirty-four miRNAs showed to be highly conserved not only among closely related species, but also between dicots and monocots (Additional File 4).

To compare miRNA abundance in different libraries, the count of each miRNA was normalized to transcripts per million (TPM), after filtering rRNA, tRNA, snRNA, snoRNA. Change in miRNA read counts between stressed and non stressed tissues in both leaves and root was recorded. On this basis, after saline treatment, 61 or 38 conserved miRNAs showed a significantly different frequency (p < 0.01), in leaves or roots, respectively (Additional File 2).

In order to identify novel artichoke miRNAs, after excluding Rfam matching sRNAs and conserved miRNAs, sRNA sequences were used to BLASTn search on artichoke ESTs and Illumina genomic sequences. Sequences containing potential new miRNAs were sent for secondary structure prediction. The fundamental criteria for a novel miRNA annotation was the occurrence of the miRNA/miRNA* duplex in a qualified stem-loop fold back structure [37]. Eight novel miRNAs were identified based on this evidence (Table 2, Additional File 3). Additional 16 sequences were also proposed to be novel artichoke miRNAs since, although not supported by the presence of miRNA*, they satisfied the secondary structure criteria and were retrieved from more than one sRNA library [37] (Additional File 2, Additional File 3). For cca-novel-7, three distinct miRNAs (cca-novel-7-1, cca-novel-7-2, and cca-novel-7-3) were considered, since they were coded by three different MIR genes. In agreement with previous results [38,39], the majority of the newly identified miRNA sequences had an uracil (U) as their first nucleotide (Table 2).

### Experimental validation and qPCR expression of selected miRNAs

**Figure 2:**
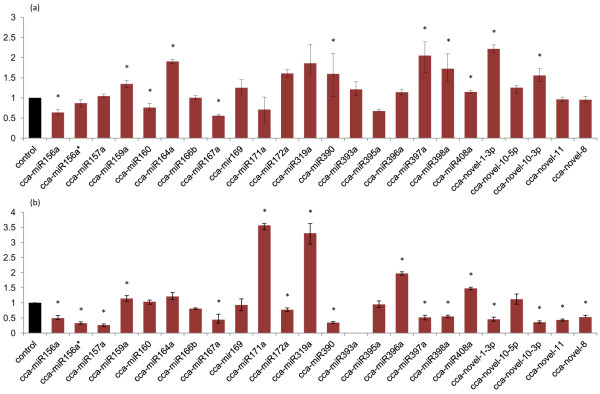
**miRNA quantitative expression**. Expression levels of selected artichoke miRNAs by means of qPCR in leaves (a) and roots (b) under control (black bar, arbitrarily set to 1) and NaCl stress (colored bars) conditions. Expression levels are given as fold change of stressed versus control tissues. Asterisks indicate significance at a p value of < 0.05. Bars refer to standard error.

qPCR was used to assay expression levels of 24 (19 conserved and 5 novel) miRNAs in artichoke leaves and roots from CP and SP using a stem-loop RT-PCR approach [40] (Figure 2). The cycle threshold (CT) value used for expression analysis for each miRNA and tissue was obtained by averaging the CT values derived from all the biological and experimental replicates. Expression changes for each miRNA were determined by arbitrarily setting the value in CP tissues as 1.0 and measuring the concentration in SP samples as fold change by means of the comparative quantitation method. Data were corrected using actin and elongation factor as housekeeping genes. To verify amplicon size, qPCR products were separated on agarose gel (Additional File 5).

Results of qPCR evidenced that a total of 11 and 17 miRNAs were significantly up or down regulated in artichoke leaves or roots, respectively. However, a two-fold or greater (ratio > 2 or < 0.5) expression difference between CP and SP was observed only for two miRNAs in leaves (cca-miR397a and cca-novel-1-3p) and for 9 miRNAs in roots, with miR171a and 319a being particularly abundant with an expression level > 3 fold change in this tissue (Figure 2). cca-miR393a was slightly up-regulated in SP leaves, while no amplification was observed in roots in our experimental conditions for both treated and untreated plants.

### Target prediction and validation

In total, we identified 565 artichoke sequences (104 ESTs and 461 Illumina genomic sequences) carrying a region of high complementarity with the previously identified miRNAs. After removing redundant sequences, only putative miRNA targets homologous to described *Arabidopsis *proteins (*E*-value less than *e*^-10^) were retained, for a total of 43 sequences, 20 matching conserved and 23 matching novel artichoke miRNAs (Additional File 6). Several cca-miRNAs (e.g. cca-miR156, 160, 164, etc.) were predicted to target known transcription factors (TFs) controlling gene expression related with plant development, morphology, and flowering time. Some of these TFs were auxin response factor involved in developmental growth, scarecrow (photomorphogenesis), SQUAMOSA promoter binding (regulation of vegetative phase change), NAC (multicellular organism development) and AP2 (organ morphogenesis; vegetative to reproductive phase transition of meristems) domain containing protein (Additional File 7). cca-miR169a targeted a nuclear transcritption factor Y involved in response to drought in *Arabidopsis *[20]. Two putative targets (for cca-miR397 and 399) were homologous to members of laccase gene family, which has been demonstrated to be involved in salt stress response [41], as well as superoxide dismutase, the target gene for cca-miR398b [42]. cca-miR403 targeted a sequence similar to AGO2, a member of the Argonaute gene family. Among novel miRNAs, predicted target genes for cca-novel-9-3p (transaldolase) and cca-novel-13-3p are involved in response to cadmium exposure (Additional File 7); miRNA cca-novel-18 was predicted to target an aspartic proteinase APA1 involved in salt stress response; cca-novel-5 target (F22C12.18) has been shown to be involved in the response to biotic (fungus) stress; cca-novel-17 targets an L-ascorbate peroxidase which plays a role in response to oxidative stress, as suggested by GO term association, while cca-novel-11 targets a sequence homologus to rubisco activase which has been demonstrated to be involved in the response to temperature variation and other stresses [43,44]. Five artichoke miRNA targets, localized in the chloroplast according to GO, were predicted to be involved in electron transport or catalytic activity, while for 7 miRNA targets no function category was retrieved (Additional File 7).

**Figure 3:**
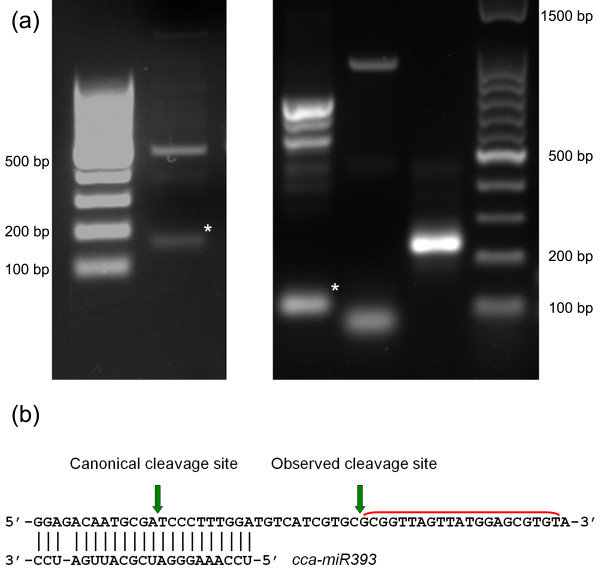
**Target validation**. (a) Separation of 5' RACE products of target genes for cca-miR-160 (left gel), and cca-miR-393, 397, 398 (right gel, from left to right). Asterisks indicate cleaved fragments. (b) Partial sequence of *TIR1 *homolog from globe artichoke (acc. No. JN382008). 21 nucleotide register phasing of sense (red) siRNAs were observed following 5' RACE analysis. Canonical and observed miR393 cleavage sites are indicated by green arrows.

For a subset of artichoke miRNAs, the cleavage of putative targets was evaluated by 5' RACE assay. For cca-miR160 target, an auxin responsive factor, a fragment of the size corresponding to the cleaved sequence was observed on agarose gel (Figure 3). Sequence of the 5' RACE cleaved product confirmed a precise slice at the miRNA binding site, between position 10 and 11. Analysis for putative targets of cca-miR397 and cca-miR398 revealed no evidence of 3' cleaved product, and only fragments corresponding to unprocessed target sequence were detected for both targets (Figure 3). Uncleaved transcripts were confirmed by sequencing.

TIR1-like (Transport Inhibitor Response 1) sequence, the putative target of cca-miR393a, was also evaluated for its cleavage site. Electrophoretic profile of RACE products highlighted a fragment of a size compatible with the miRNA cleavage (Figure 3). However, when we cloned and sequenced eight clones carrying the gel purified PCR product, we observed a fragment cleaved in a different position from the expected one. All sequences were sliced 21 nucleotides downstream the canonical miRNA-target duplex region (Figure 3). Furthermore, in order to find any other sRNA matching this cleaved TIR1-like sequence, we aligned all our sRNA Illumina reads on the cleaved target and found 21, 22-nt long sRNAs mapping on both sense and antisense strands of artichoke TIR1-like sequence, downstream the cca-miR393 predicted cleavage site, with a particular abundance of a sequence mapping in position 190-210 (sense strand). These sRNA reads were detected in libraries from stressed and non stressed leaves but not in root tissues.

## Discussion

The existence of orthologous conserved miRNAs in a plant species can be easily confirmed by using computational approaches, due to high conservation of many mature miRNAs across a wide range of plant species [45,46], or by traditional Sanger sequencing. Yet, following these strategies, species-specific miRNAs, which are generally poorly expressed, cannot be easily detected. Thousands of miRNAs from several plant species have been to date described and partly characterized. For artichoke, however, miRNAs have not been released yet.

The genome of globe artichoke has been scarcely characterized so far, and sequences available in the public databases are limited to about 36,000 ESTs and just a few genomic and mRNA sequences. In order to isolate and analyze the miRNAome of artichoke, we used a deep sequencing approach on four sRNA libraries obtained from two tissues, leaves and roots, in standard conditions and under saline treatment. Like for several other plant species, 24-nt sRNAs were the most abundant size class; this result is consistent with the typical size of Dicer digestion products [6,47-49]. We compared the obtained sRNA sequences not only to the public artichoke ESTs, but also to a quite large set of proprietary Illumina sequences representing about 2.3 × coverage of the artichoke genome. This procedure allowed us to discover and describe, for the first time, a total of 122 artichoke miRNAs including 98 known and 24 novel ones. Conserved miRNAs were detected in very stringent conditions, by considering only perfect matching sequences, without permitting any mismatch. The characteristic stem-loop secondary structure of pre-miRNA allows to distinguish miRNAs from other small RNAs such as short-interfering RNAs (siRNAs) [37]. For novel miRNAs, the primary criterion for annotation is the finding of the corresponding miRNA* in sequencing data sets. In the absence of the miRNA*, the discovery of new miRNAs can be supported by the identification of the stem-loop structure together with the presence in multiple, independent libraries [37].

It is well known that most MIR genes map in regions annotated as intergenic or non protein coding genes. However, miRNA precursors complementary to protein coding genes or matching to introns can also be found [15]. Since the artichoke genome is only partially known (see above), we could not study the genomic organization of artichoke miRNAs.

Although estimation of expression levels can be performed from sequencing reads, we validated levels of expressions for a number of conserved and novel artichoke miRNAs by qPCR, since no biological or experimental replicates had been used for sequencing. Results were not always consistent between the two approaches. We will therefore mainly discuss expression levels obtained from qPCR experiments. We used the comparative quantitation method which does not require any extra RT-PCR reactions to calculate PCR efficiencies, is cheaper, less time consuming and uses fewer reagents compared to the more commonly used comparative threshold cycle method [50,51]. In general, the magnitude of variation in expression levels for artichoke miRNAs after salt stress seemed to be more pronounced in roots than in leaves.

Some of the artichoke conserved miRNAs here validated had already been tested in other species in response to salt stress. Most studies are referred to *Arabidopsis*, where miR159 and 319 were up-regulated following saline treatment, as in both artichoke tissues; in other cases there is a good correspondence only with one of the artichoke tissues (e.g. miR397 and 398), whereas, for some other miRNAs (miR156 and 167) the regulation after salt stress was opposite in *Arabidopsis *compared to artichoke [[19], and references therein]. However, also some miRNAs from other plant species have displayed a response to salt treatment different from that of *Arabidopsis*; this is the case of maize (miR156, and 396), *Populus *(miR398), and *Medicago truncatula *(miR396) [15]. Interestingly, in *M. truncatula*, miR390 was down regulated in roots, like in artichoke.

Several evidences have shown that most plant miRNAs function by either perfectly or near-perfectly binding to complementary sites on their target mRNA sequences [52]. This provides a powerful way to identify potential targets simply by aligning and comparing miRNAs with a set of available sequences. Since few protein-coding genes have been reported for artichoke so far, all ESTs and proprietary Illumina genomic sequences were used for target prediction. Detection of artichoke putative targets was performed in stringent conditions, and only genes matching to described *Arabidopsis *proteins were considered. Most targets identified in this study were TFs, as also observed for other species. In fact, many studies have demonstrated, by experimental and computational approaches, that miRNAs target prevalently TFs involved in plant development control. In addition, many other predicted artichoke targets were homologous to proteins involved in plant response to several abiotic and biotic stresses; this is the case also for putative targets of novel artichoke miRNAs (Additional File 6, Additional File 7).

As for conserved miRNAs, cca-miR156 possessed different isoforms recognizing distinct targets (Additional Files 6). As proposed for miR169a and its NFY-A TF targets for drought response and abscisic acid signaling, different isoforms of miRNAs might regulate distinct target genes and define complex specificities at spatio-temporal level [15,20].

For some artichoke miRNAs regulated following saline stress, targets could not be identified possibly because the artichoke genome is still largely unexplored. However, members of miR164 and 172 families targeted NAC and AP2 TFs, respectively; miR397 and 399 families targeted members of the laccase gene family, and miR398 family, a superoxide dismutase. Laccases are multicopper-containing glycoproteins, widespread in plants. Many physiological functions have been associated with laccases, particularly lignin biosynthesis [53] and formation of proanthocyanidin or tannin [54]. Moreover, laccase, like other polyphenol oxidases, is believed to be responsible for polymerization of phenolic compounds which protect plants from pathogen and insect attack [55]. It has been demonstrated that the transcript level of laccase genes is enhanced by high concentrations of NaCl in tomato, maize, and *Arabidopsis *roots [41,56,57], suggesting that an increase in laccase transcript level in roots under salinity stress could be a universal response in plants. In artichoke, a down-regulation of miR397a in roots might possibly lead to a higher expression of laccase in this organ, particularly involved in the response to salt stress.

miR398 expression level has been shown to be affected by salt stress treatment, as well as by other abiotic and biotic stresses [42]. We found a contrasting behavior in leaves (up-regulation) compared to roots (down-regulation). When *Arabidopsis *seedlings were analysed after imposing NaCl treatment, a slight increase in miR398 expression was observed, accompanied by a low decrease in the expression of its target, superoxide dismutase. However, it has also been shown that distinct members of the superoxide dismutase family can display a different behavior following salt stress [58].

miR403 validated target is AGO2 mRNA [59,60]. This interaction miRNA/mRNA is likely to be involved in a multiple layer RNA-mediated defense and counter-defense in the interactions between plants and their viruses, as recently reported in Arabidopsis [61]. In this model, AGO1 acts in the first layer in defense against viruses, while AGO2 is involved in the second layer limiting virus accumulation. When the primary AGO1-mediated mechanism is overcome by viral suppressors of silencing the second layer is activated because AGO2 is no longer repressed by AGO1 via miR403.

5' RACE approach can unambiguously diagnose one of the mechanisms of action of miRNAs, by endonucleatic cleavage at the pairing site of miRNA with its target [2] and has been successfully used for both total and poly(A)-selected RNA templates [62]. However, in our study, not all the targets tested showed the expected cleaved fragment, possibly because translational repression of targets might be the preferred mechanism of action for some miRNAs. In fact, notwithstanding almost perfect complementarity between miRNA and its target sequence in plants, it has been demonstrated that likewise in animals, non-cleaving repression is a possible mechanism also in the plant kingdom [63]. Moreover, it has been hypothesized that at times, the mechanisms of slicing and translational repression might be spatially or temporally separated [2].

For artichoke miR393 putative target, TIR1, we observed a 21 nucleotide shorter cleaved sequence after 5' RACE analysis. As previously reported in *Arabidopsis*, this can be attributed to secondary siRNA in the 21-nucleotide register with the cleavage site for miR393 [64]. In some cases, in fact, after miRNA-directed cleavage, sliced target RNAs can generate secondary phased siRNAs. After cleavage, the targeted RNA is converted into dsRNA by RNA-dependent RNA polymerases (RDRs), which is then sliced into secondary siRNAs by DICER-LIKE (DCL) nucleases [65]. Most miRNA-targeted RNAs do not generate secondary siRNAs, suggesting the existence of additional determinants of secondary siRNA production. A "two-hit" model to explain this secondary effect of miRNA mediated cleavage is based on the evidence that two miRNA target sites are critical for TAS3 tasiRNA production between the two sites [66]. This model, however, does not explain secondary siRNA triggering by single site miRNA targets as for miR393 targeting TIR1 transcript. Recently, it has been suggested that, in *Arabidopsis*, secondary siRNA triggers are represented as 22-nt miRNAs rather than the canonical 21-nt long miRNAs [67,68]. miR393 target TIR1-like protein is well conserved in many plant species and is involved in the response to auxin stimulus in concert with the Aux/IAA transcriptional repressors [69]. siRNAs from TIR1 were shown to be in 21-nucleotide register with the upstream miRNA cleavage site predicted by the sequence of miR393. Here we reported the same behavior for artichoke TIR1-like sequence, suggesting that this mechanism can be a general one, diffused in all plant species.

## Conclusions

In conclusion, we analysed the miRNAome of globe artichoke, detecting conserved and novel miRNAs for artichoke. Some of them were differentially regulated in leaves or roots in standard conditions or after imposing saline stress. Putative targets were identified and some of them were validated for their cleavage site. Interestingly, we could demonstrate that also in artichoke, TIR1 gene, the putative target for miR393, generates secondary siRNAs.

## Methods

### Plant material and growth conditions

Artichoke seeds, cultivar Harmony, were obtained from Nunhems (Haelan, NL) and germinated in Petri dishes on moist filter paper. After germination, seedlings were grown in a greenhouse with 16 h light/8 h dark at 22°C. In order to avoid unintended abiotic stresses, plants were watered with nutrient solution instead of simple water.

Four week-old artichoke seedlings were subjected to salt stress by dipping roots in 250 mM NaCl solution for 8 h. Leaves and roots from stressed and control plants were collected and immediately stored in liquid nitrogen until RNA extraction.

### Small RNA extraction and library preparation

sRNAs were isolated from 100 mg of tissue using the mirPremier microRNA Isolation Kit (Sigma-Aldrich, St. Louis, MO, USA) and RNA quality was assessed by agarose gel according to manufacturer's instructions.

sRNA library construction, cluster generation and deep sequencing was performed following Illumina protocols (Preparing Samples for Small RNA Sequencing Using the Alternative v. 1.5 Protocol, Illumina Inc., San Diego, CA, USA). Briefly, 5 μl sRNA fraction from artichoke tissues were ligated to the v. 1.5 sRNA 3' adapter (AUCUCGUAUGCCGUCUUCUGCUUG) using T4 RNA Ligase 2, truncated (New England BioLabs, Ipswich, MA, USA) at 22°C for 1 h. sRNAs from previous step were ligated to the SRA 5' adapter (GUUCAGAGUUCUACAGUCCGACGAUC) with T4 RNA ligase (New England BioLabs) at 20°C for 1 h. In order to synthesize first-strand cDNA, 5'/3' adapter-ligated sRNAs were reverse transcribed using SuperScript II Reverse Transcriptase (Invitrogen Paisley, UK) at 44°C for 1 h. A 12 cycles cDNA amplification was performed to selectively enrich samples of fragments with both adapters ligated, using Phusion DNA Polymerase (Finnzymes Oy, Finland) with the primer combination GX1 (CAAGCAGAAGACGGCATACGA), GX2 (AATGATACGGCGACCACCGACAGGTTCAGAGTTCTACAGTCCGA) according to Illumina protocol. cDNA fragments were separated on PAGE gel and purified after size selection (for 22-30 nt). The purified library was quantified using Bioanalyzer 2100 (Agilent Technologies, Santa Clara, CA, USA) and used for cluster generation on a single read flowcell. All libraries were sent for sequencing on Illumina Genome Analyser II (GAII) in a 36-cycle run, at the Istituto di Genomica Applicata (IGA), Udine, Italy.

### Sequencing data analysis and miRNA identification

After trimming off the adaptors, unique sequences between 16 and 30 nucleotides in length and counted three or more times in at least one library were used to further analysis. These sRNA sequences were compared to known plant miRNAs from the miRBase Sequence Database, release 17.0, using a custom script, in order to identify conserved miRNAs in artichoke. Only identical matching sequences to currently known plant miRNAs were considered as conserved artichoke miRNAs.

To identify miRNA precursor sequences, artichoke conserved miRNAs were used to BLASTn search against artichoke ESTs in NCBI and against a set of proprietary genomic sequences corresponding to a 2.3 × coverage of the artichoke genome (data not shown). ESTs and genomic sequences with four or less mismatches with conserved artichoke miRNAs were considered as competent sequences and used for fold-back structure prediction by Mfold program (http://mfold.rna.albany.edu/?q=mfold/RNA-Folding-Form[70]). A region of at least 70 nucleotides containing the mature miRNA (the putative pre-miRNA) was extracted from each competent artichoke sequence and checked for the following criteria: 1) folding into an appropriate stem-loop hairpin secondary structure with the mature miRNA sequence on one arm of the hairpin; 2) presence of no more than 6 mismatches between the mature miRNA sequence and the opposite miRNA*; 3) minimal free energy index (MFEI) of predicted secondary structures higher than 0.67; 4) 30-70% A + U contents. These criteria significantly reduced assignment of false positives.

To identify novel miRNAs from artichoke, all 18-25 nucleotides long sRNA sequences were used for BLASTn search against ESTs and genomic sequences after discarding known miRNAs and sequencing matching rRNA, tRNA, snRNA and snoRNAs from Rfam database (http://rfam.sanger.ac.uk/). The sRNAs possessing stem-loop precursors were regarded as putative non-conserved miRNAs. We searched miRNA* sequences (complementary to miRNA in the precursor molecule) in the sRNA libraries.

Genomic precursor sequences for both conserved and novel miRNAs were submitted to NCBI database (accession numbers JN381965- JN381978, JN381980-JN382005, JQ029164, JQ029165).

For each library, the count of each miRNA was normalized to transcripts per million (TPM). The significance of differences in miRNA frequency between growing conditions was set at a p value < 0.01 using a χ^2 ^test.

### Quantitative qPCR expression

Total RNA was reverse transcribed using SuperScript III Reverse Transcriptase (Invitrogen, US) followed by DNAse treatment. miRNAs were detected using stem-loop RT-PCR method [40]; primers sequences are reported in Additional File 8. For housekeeping genes, actin and elongation factor, cDNA was reverse transcribed using two specific reverse primers using the same protocol as for miRNAs.

For qPCR analysis, each reaction contained 20 mM Tris-HCl (pH 8.4), 50 mM KCl, 1.5 mM MgCl2, 0.1 mM dNTP mixture, 0.2 μM of each primer: a miRNA-specific forward primer and a universal reverse primer (Additional File 8); 1 U of Taq DNA Polymerase Recombinant (Invitrogen), 1 μl of 20x Evagreen dye (Biotium, Inc. Hayward, CA, USA), in a final volume of 20 μl. PCR conditions were: 94°C for 3 min, followed by 40 cycles of 94°C for 15 sec and 60°C for 1 min. Reactions were performed in a Rotor Gene 6000 (Corbett Mortlake, AUS) and dissociation curves of PCR products were carried out in order to assess the specificity of amplification reactions. Three biological replicates for each sample were analyzed; each experiment included a no-template control and was repeated three times. As housekeeping genes, artichoke actin and elongation factor [30,71] were used, since their expression values after salt stress showed no significant changes compared to the control. Real-time products were visualized on agarose gel.

Data were analyzed using the "Comparative Quantitation" software supplied by Corbett Research for the Rotorgene. This method for qPCR data analysis, calculates the efficiencies of each transcript for each individual PCR reaction and is based on the second differential maximum method [72] to calculate single reaction efficiencies. These data were applied to the Relative Expression Software Tool (REST 2009, [51]) to assess significance by a randomization test. Statistical significance was accepted at a p value of < 0.05.

### Target prediction and validation

Novel and conserved artichoke miRNAs were used as query sequences for BLASTn search against all artichoke ESTs in NCBI and against proprietary Illumina genomic sequences [34]. Alignments between each miRNA and its putative mRNA target were evaluated following parameters based on complementarity between them: 1) no more than four mismatches were allowed between the mature miRNA and its target site; 2) no more than one mismatch was allowed at nucleotide positions 1-9; 3) no more than two consecutive mismatches were allowed; 4) no mismatches were allowed at positions 10 and 11 [73]. The extracted sequences (EST and genomic ones) corresponding to artichoke putative miRNA targets were used for a tBLASTx search against all *Arabidopsis thaliana *non-redundant proteins from the NCBI database to identify putative gene homologs. Similarities with an E-value lower than e^-10 ^were considered as positive hits. Gene Ontology (GO) terms associated to *Arabidopsis *homologs of putative artichoke miRNA targets were identified using the BLAST search tool of AmiGO (http://amigo.geneontology.org/cgi-bin/amigo/go.cgi).

Genomic precursor sequences for artichoke miRNA targets were submitted to NCBI database (accession numbers JN382006-JN382024).

To validate target genes, a 5' RACE assay was performed using the 5' RACE System for Rapid Amplification of cDNA Ends (Invitrogen) on total RNA extracted with TRizol^® ^reagent (Invitrogen) from artichoke leaves, following the manufacturer instructions. Gene-specific reverse primers were designed on the basis of artichoke putative target sequences, and used in combination with the 5' RACE adapter primer to amplify transcripts (Additional File 9). PCR products were sequenced to confirm cleavage at the expected site.

## Authors' contributions

GS and DP conceived the work; FC was responsible for small RNA sequencing; DD conducted the experimental procedure for detection and analyses of miRNAs and target genes; GS and DD analyzed results; GS supervised the work. All authors read and approved the final manuscript.

## Supplementary Material

Additional file 1 **Small RNA categories**. Classification of small RNAs in artichoke tissues and their relative abundance (expressed as % of reads in each library). Click here for file

Additional file 2 **Artichoke miRNAs**. Conserved and novel miRNAs from artichoke, and their count as transcripts per million (TPM) in four sRNA libraries. Click here for file

Additional file 3 **Folding miRNA structures**. Secondary structures of conserved and novel miRNAs from artichoke. Click here for file

Additional file 4 **miRNA conservation**. Conservation of known artichoke miRNAs across 46 plant species. Click here for file

Additional file 5 **miRNA electrophoretic profiles**. Agarose gel separation of qPCR products of selected artichoke miRNAs. Click here for file

Additional file 6 **Identification of putative targets for conserved and novel artichoke miRNAs**. Function was inferred using artichoke sequences as query for BLASTx searches against *Arabidopsis thaliana *proteins Click here for file

Additional file 7 **Target annotation**. Artichoke miRNA targets annotated according to GO terms for *Arabidopsis *homologous proteins. Click here for file

Additional file 8 **qPCR primers**. Reverse transcription (RT), forward and reverse primers used for artichoke miRNA validation and quantitative real-time PCR analyses. Click here for file

Additional file 9 **Target validation primers**. Gene specific primers (GSP) used for reverse transcription (GSP1) and PCR amplifications (GSP2 and GSP3) for the validation of miRNA targets. Click here for file
