# Supplementary material for: The miRNAome of globe artichoke: conserved and novel micro RNAs and target analysis
Source: BMC Genomics. 2012 Jan 24;13:41. doi: 10.1186/1471-2164-13-41 (PMC3285030; doi:10.1186/1471-2164-13-41)
Supplement: Additional file 1 — Small RNA categories. Classification of small RNAs in artichoke tissues and their relative abundance (expressed as % of reads in each library). [file 1471-2164-13-41-S1.DOC]

**Additional File 1** Classification of small RNAs in artichoke tissues and their relative abundance (expressed as % of reads in each library)

| Category | Leaves | | Roots | |
| --- | --- | --- | --- | --- |
|  | CP (%) | SP (%) | CP (%) | SP (%) |
| snRNA | 1.04 | 0.66 | 1.03 | 0.52 |
| snoRNA | 1.30 | 0.93 | 1.01 | 0.59 |
| rRNA | 7.00 | 11.14 | 13.19 | 34.04 |
| tRNA | 3.90 | 4.78 | 2.34 | 5.55 |
| total sRNAs matching Rfam | 16.16 | 19.88 | 20.32 | 44.72 |

CP: control plant; SP: stressed plant
