# Supplementary material for: The miRNAome of globe artichoke: conserved and novel micro RNAs and target analysis
Source: BMC Genomics. 2012 Jan 24;13:41. doi: 10.1186/1471-2164-13-41 (PMC3285030; doi:10.1186/1471-2164-13-41)

Additional File 3 Secondary structures of conserved and novel miRNAs from artichoke

cca-miR156a
cca-miR156a*

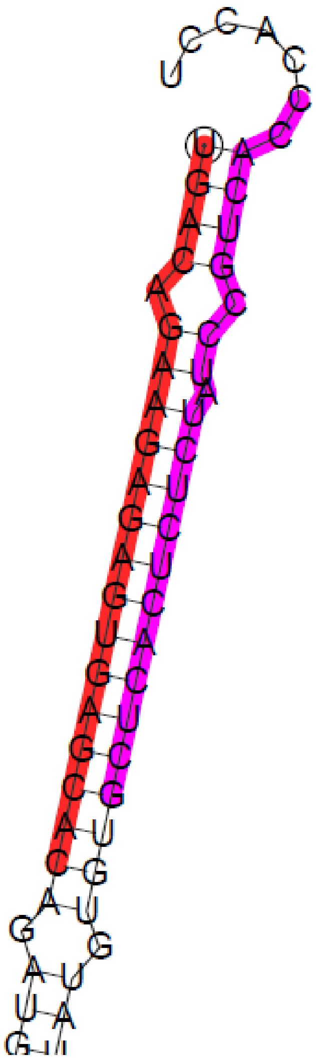

cca-miR156b

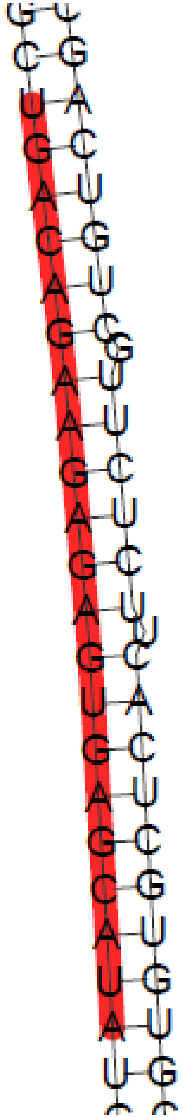

cca-miR157a

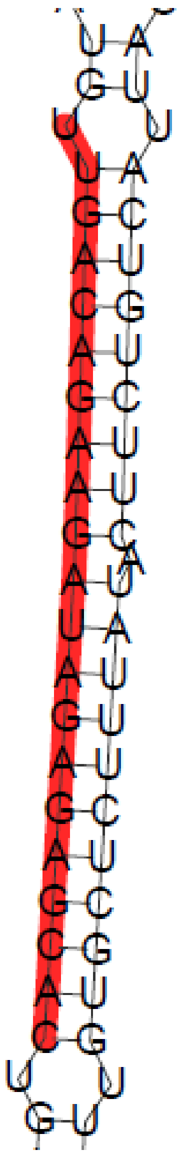

cca-miR160-1

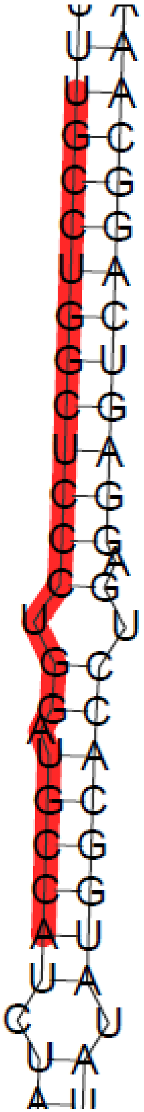

cca-miR160-2

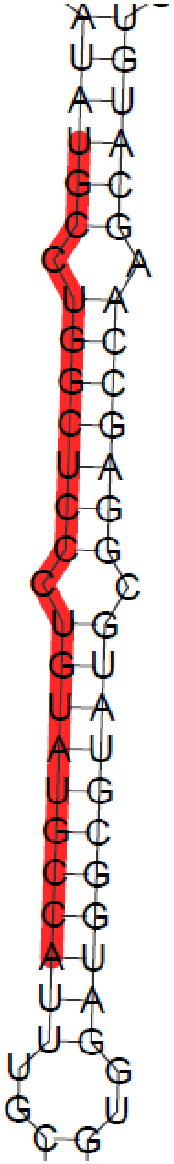

cca-miR164a

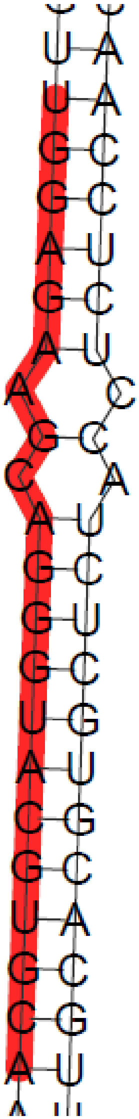

cca-miR166d
cca-miR166d*

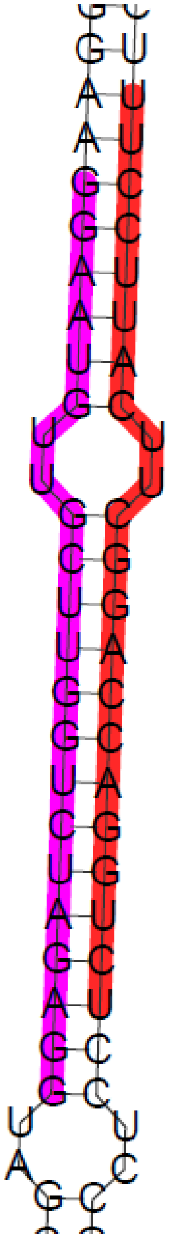

cca-miR167a

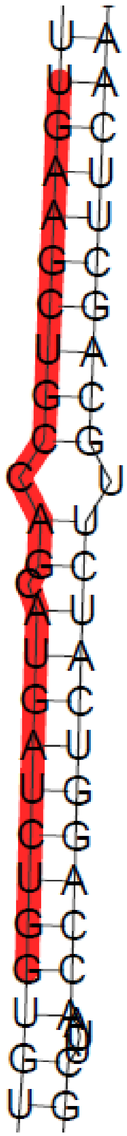

cca-miR168a

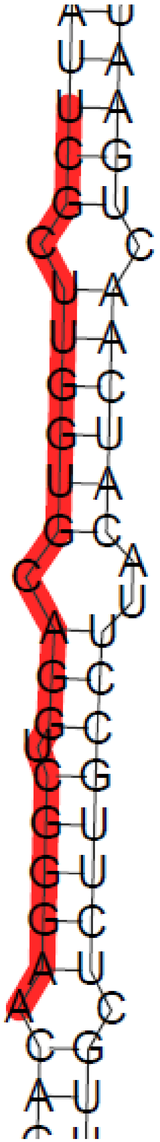

cca-miR169a-1

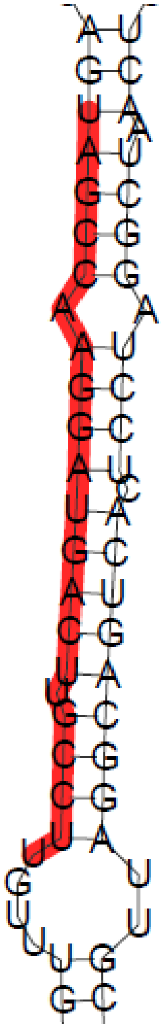

cca-miR169a-2

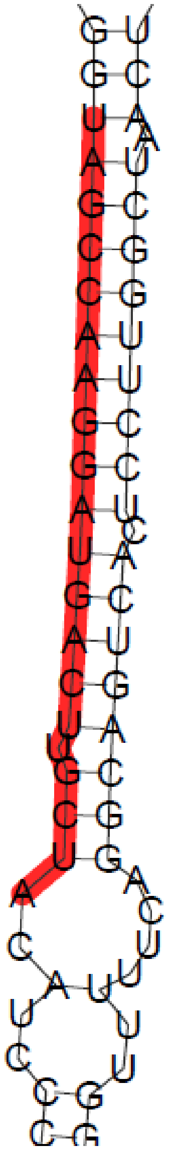

cca-miR171a

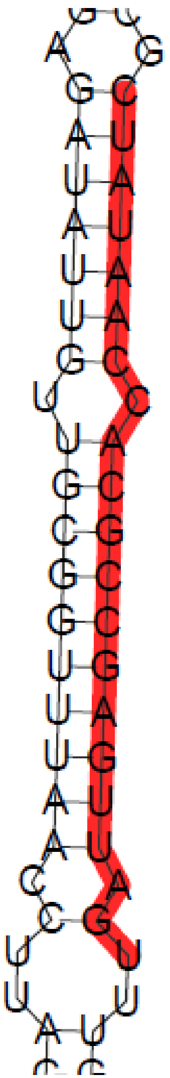

cca-miR172a

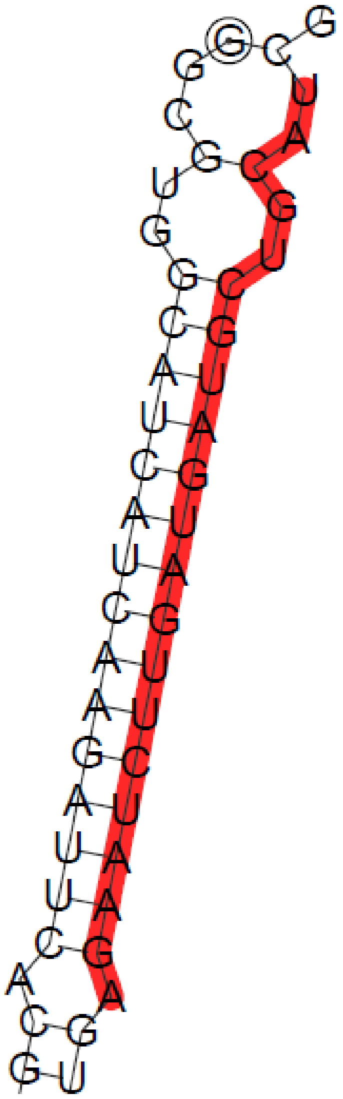

cca-miR319c

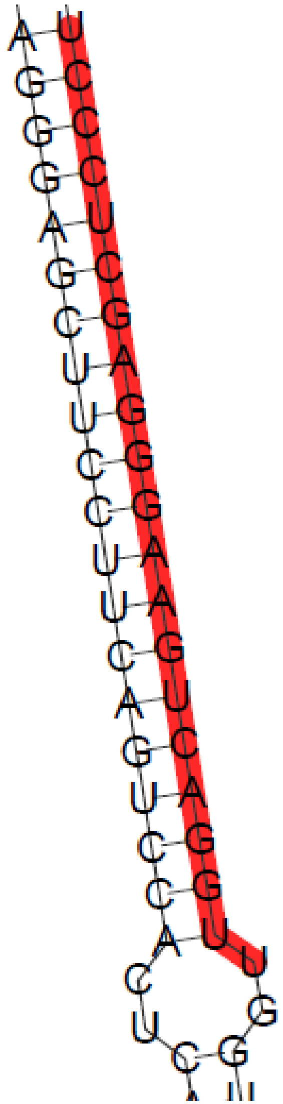

cca-miR390

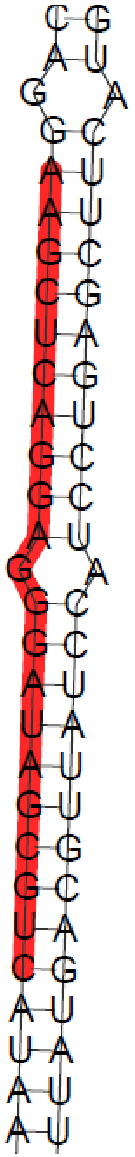

cca-miR393a

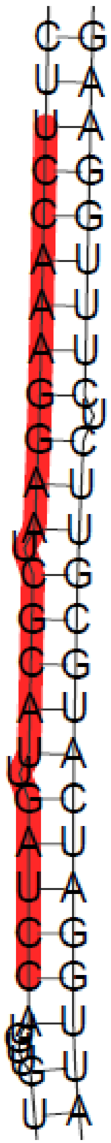

cca-miR394

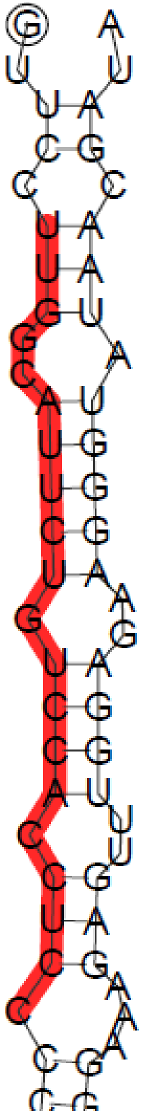

cca-miR395a

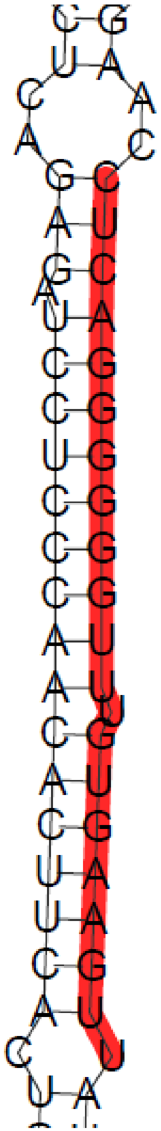

cca-miR395b-1

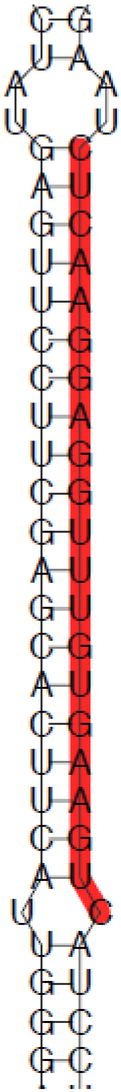

cca-miR395b-2

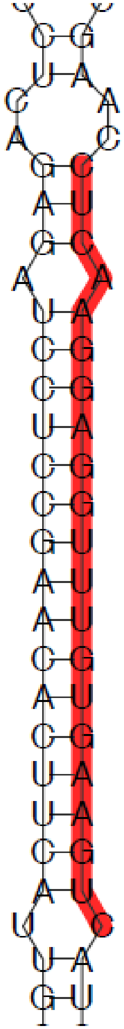

cca-miR396a
cca-miR396a*

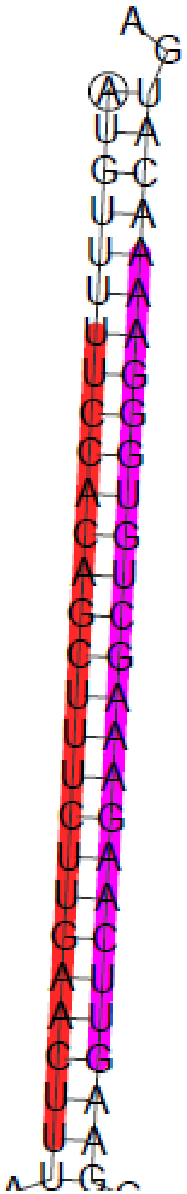

cca-miR396b

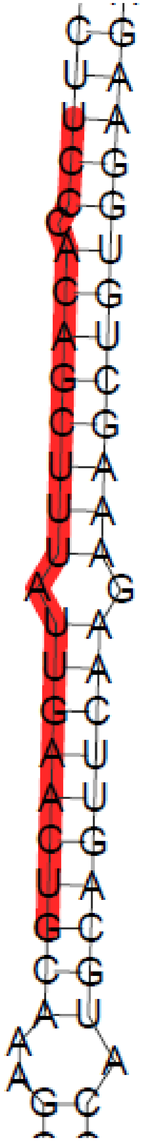

cca-miR398a

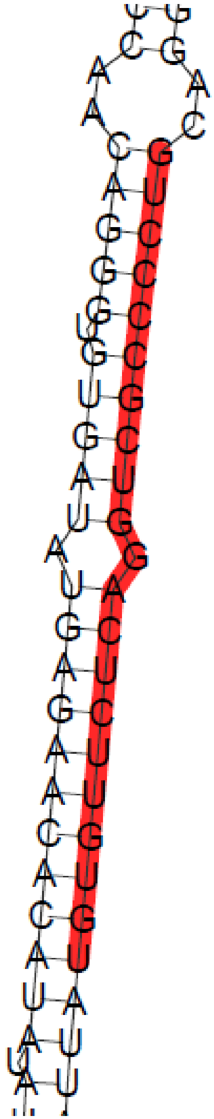

cca-miR399a

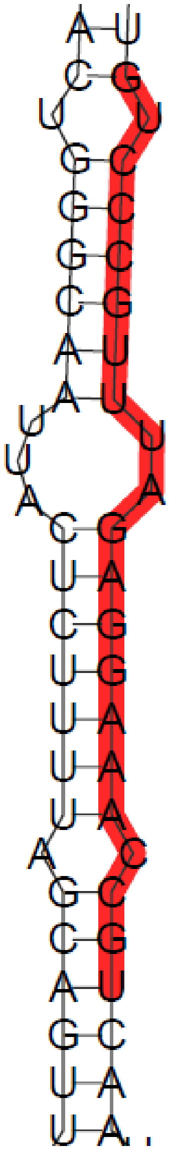

cca-miR408a

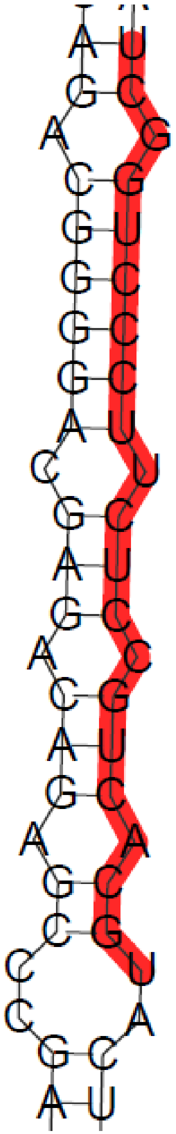

cca-novel-1-5p
cca-novel-1-3p

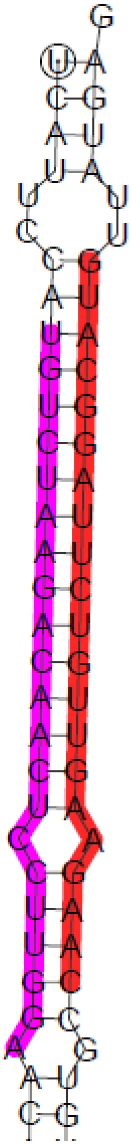

cca-novel-2

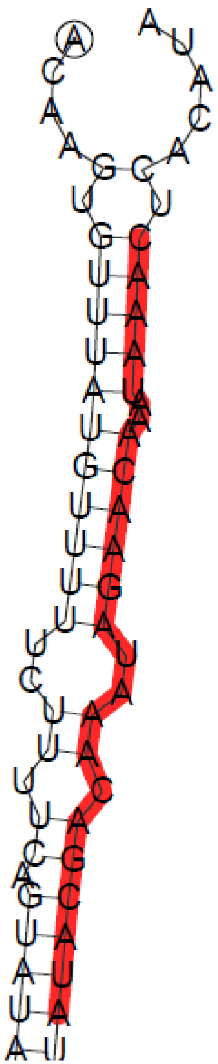

cca-novel-3

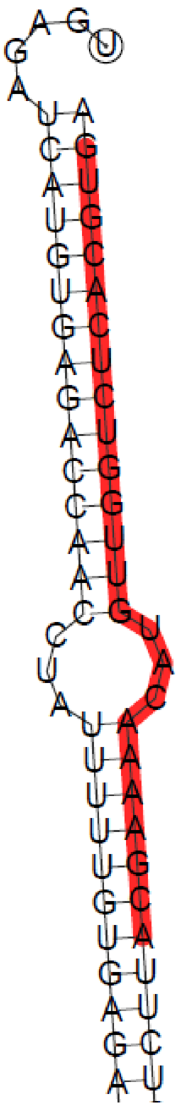

cca-novel-4-5p
cca-novel-4-3p

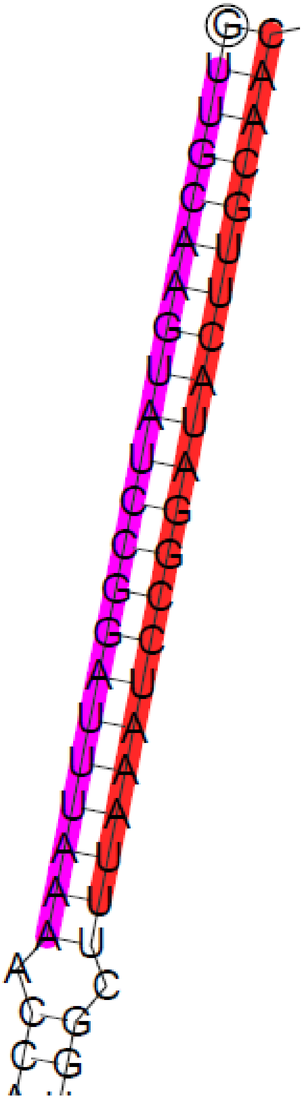

cca-novel-5

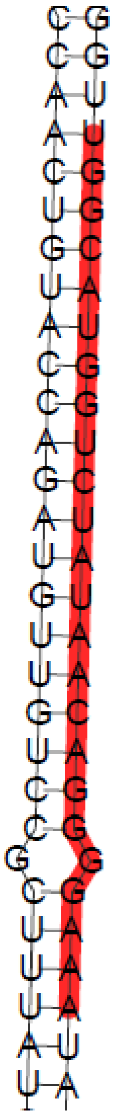

cca-novel-6

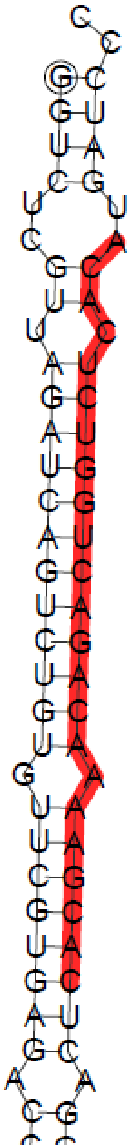

cca-novel-7-1

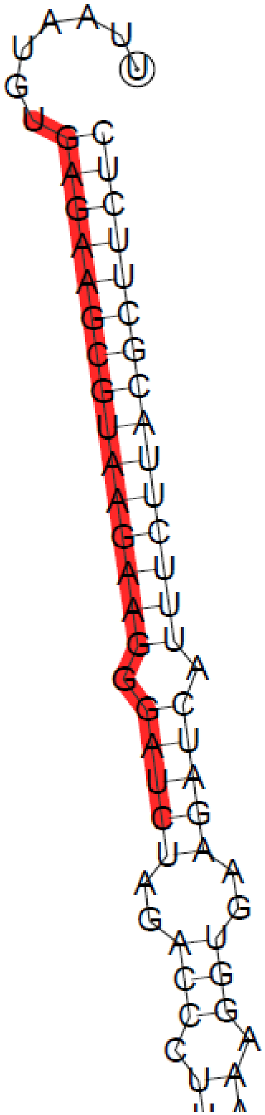

cca-novel-7-2

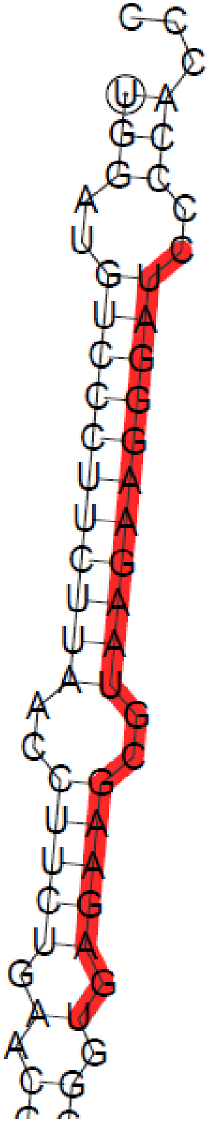

cca-novel-7-3

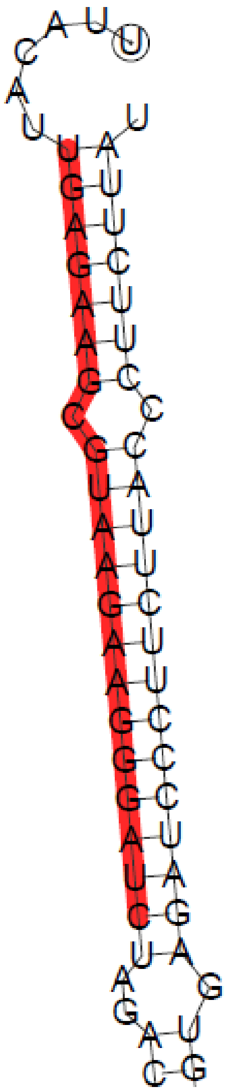

cca-novel-8

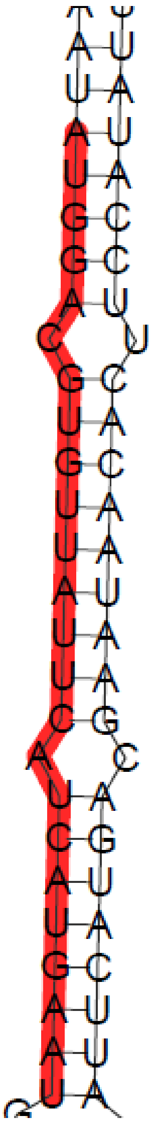

cca-novel-9-5p
cca-novel-9-3p

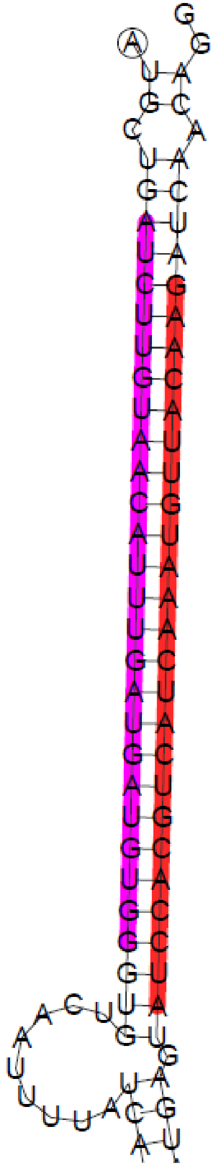

cca-novel-10-5p
cca-novel-10-3p

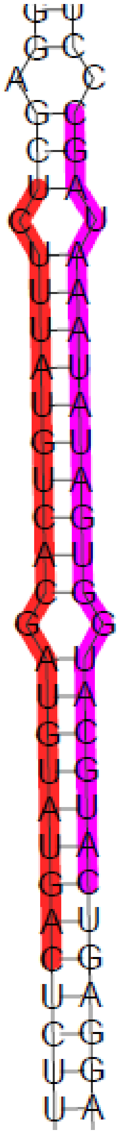

cca-novel-11

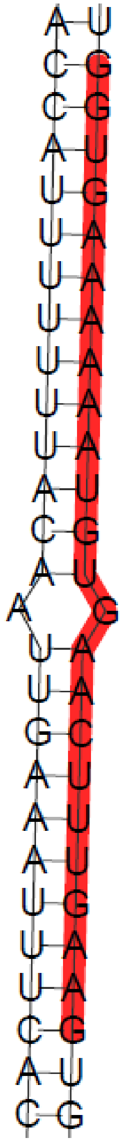

cca-novel-12

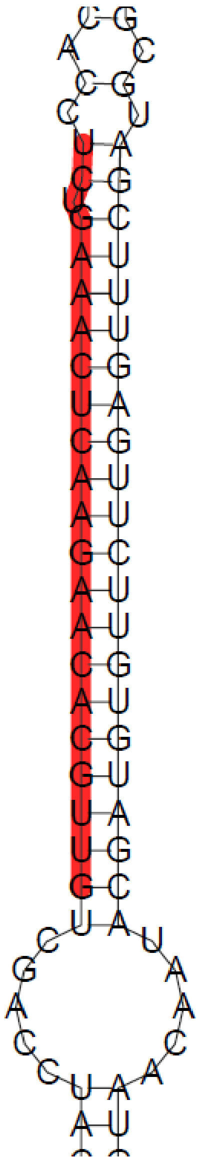

cca-novel-13

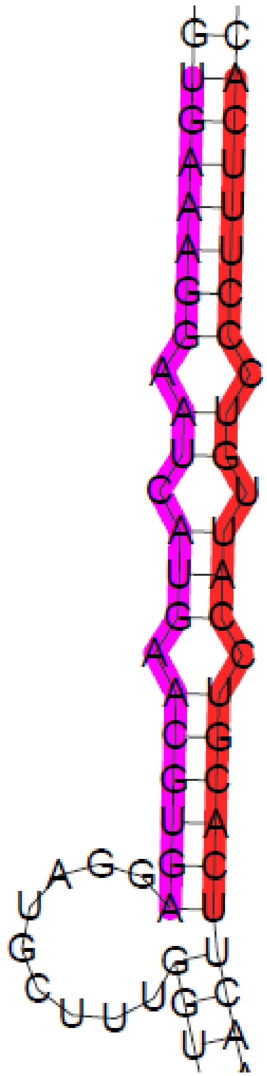

cca-novel-14

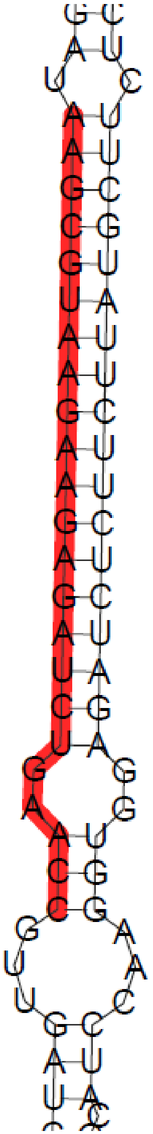

cca-novel-15

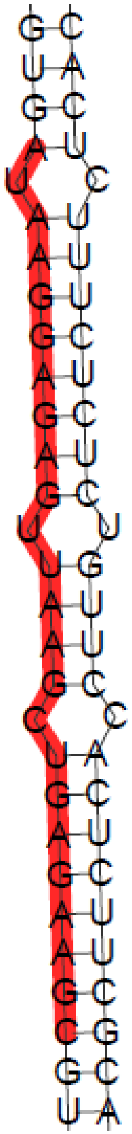

cca-novel-16-5p
cca-novel-16-3p

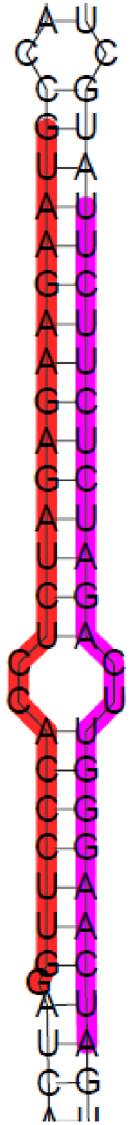

cca-novel-17

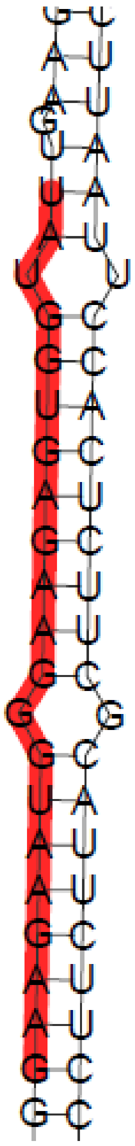

cca-novel-18

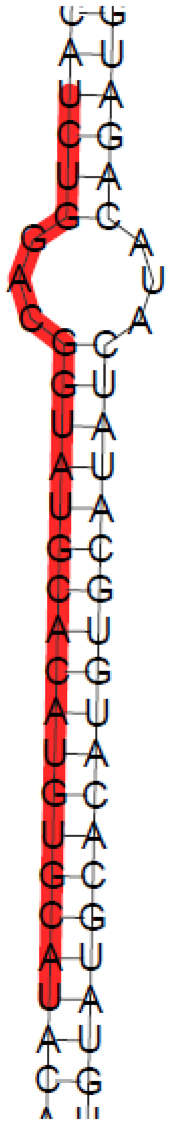

cca-novel-19

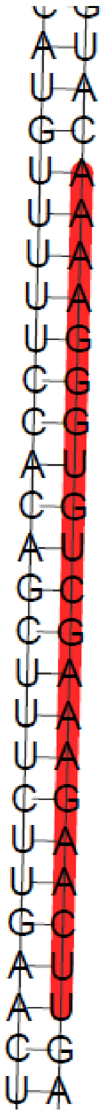

cca-novel-20-5p
cca-novel-20-3p

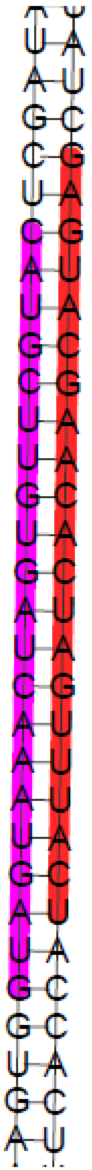

cca-novel-21

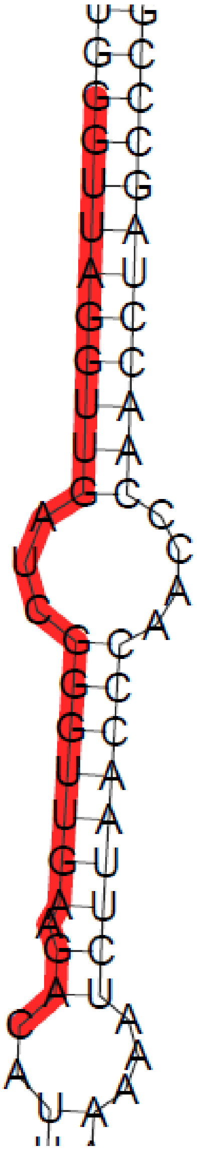

cca-novel-22-5p
cca-novel-22-3p

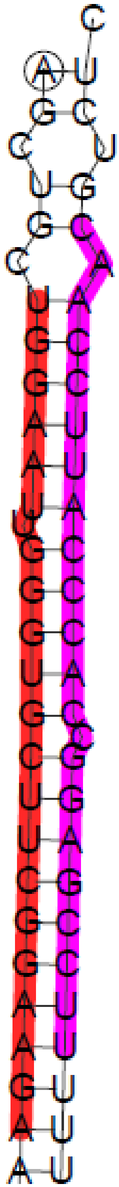

Supplement: Additional file 3 — Folding miRNA structures. Secondary structures of conserved and novel miRNAs from artichoke. [file 1471-2164-13-41-S3.PDF]
