# Supplementary figures and images for: The miRNAome of globe artichoke: conserved and novel micro RNAs and target analysis
Source: BMC Genomics. 2012 Jan 24;13:41. doi: 10.1186/1471-2164-13-41 (PMC3285030; doi:10.1186/1471-2164-13-41)

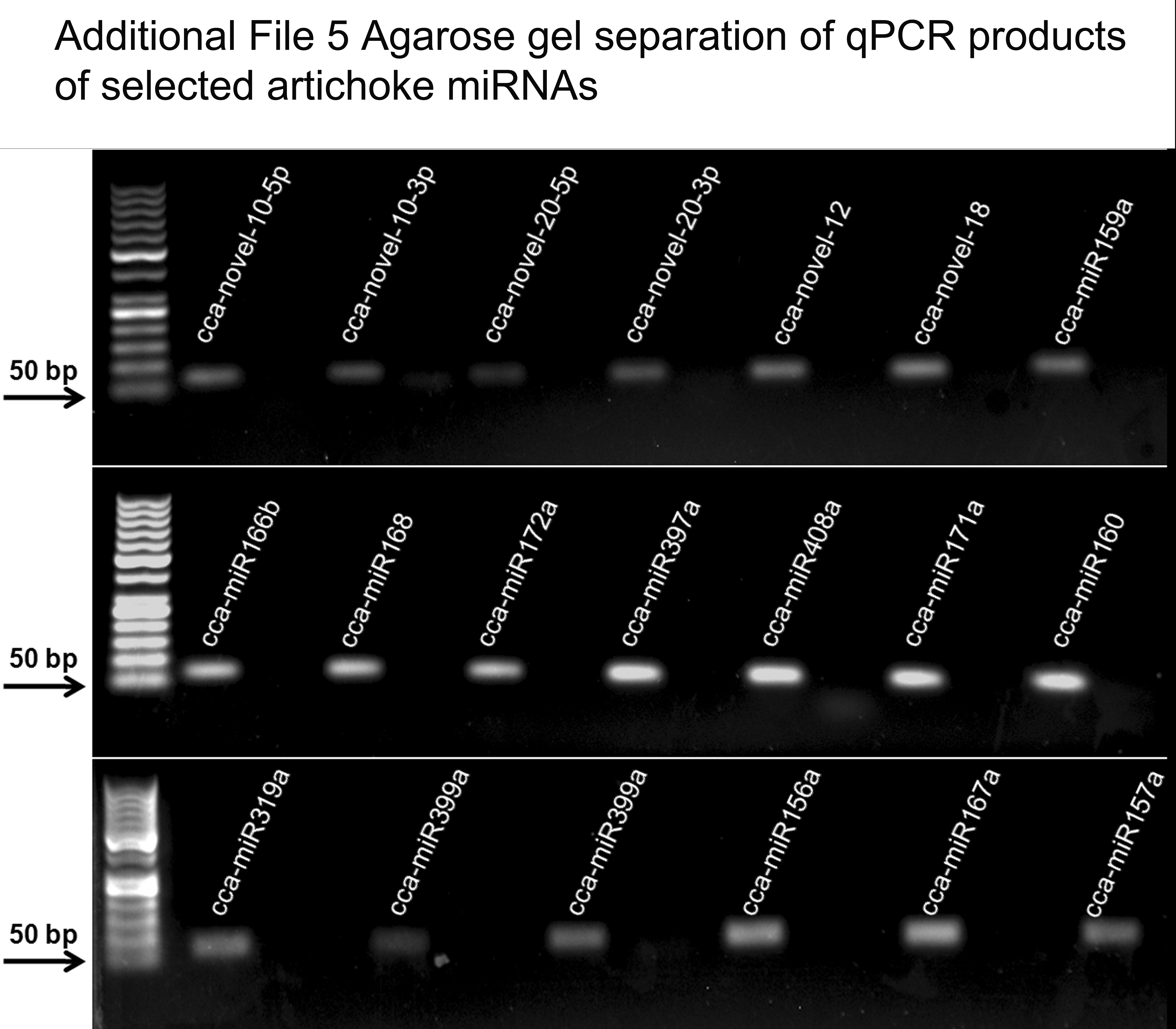

Supplement: Additional file 5 — miRNA electrophoretic profiles. Agarose gel separation of qPCR products of selected artichoke miRNAs. [file 1471-2164-13-41-S5.TIFF]
