# Supplementary material for: The miRNAome of globe artichoke: conserved and novel micro RNAs and target analysis
Source: BMC Genomics. 2012 Jan 24;13:41. doi: 10.1186/1471-2164-13-41 (PMC3285030; doi:10.1186/1471-2164-13-41)
Supplement: Additional file 6 — Identification of putative targets for conserved and novel artichoke miRNAs. Function was inferred using artichoke sequences as query for BLASTx searches against Arabidopsis thaliana proteins [file 1471-2164-13-41-S6.DOC]

**Additional File 6** Identification of putative targets for conserved and novel artichoke miRNAs. Function was inferred using artichoke sequences as query for BLASTx searches against *Arabidopsis thaliana*  proteins

| miRNA | Target | Target gene function | Score/*E*-value |
| --- | --- | --- | --- |
| cca-miR156h, k, o^1^ | JN382010 | CAG25585- putative squamosa-promoter binding protein | 54/4e-12 |
| cca-miR156a, b, d, e, f, g, h, k, l, m, o^1^ | GE592883.1 | AAM67271- putative squamosa-promoter binding protein | 136/6e-32 |
| cca-miR156a, d, e, f, g, h, l, m, n, o^1^/ cca-miR157a | GE596188.1 | CAB56584- squamosa promoter binding protein-like 4 | 111/1e-24 |
| cca-miR156i | GE610452.1 | NP_850509- FMN binding protein | 307/2e-83 |
| cca-miR156j | GE580676.1 | NP_564033- Protein phosphatase 2A, regulatory subunit PR55 | 357/1e-98 |
| cca-miR157b | GE600219.1 | 3RHB_A- Chain A, Crystal Structure Of The Apo Form Of Glutaredoxin C5 | 175/9e-44 |
| cca-miR159 | JN382009 | AAF79778- T32E20.1 | 85/2e-17 |
| cca-miR160 | GE577464.1 | NP_180402- auxin response factor 10 | 229/5e-60 |
| cca-miR164a | GE610743.1 | NP_568182- NAC domain containing protein 80 | 312/6e-85 |
| cca-miR166e, f, g^1^ | GE579337.1 | NP_001077702- homeobox-leucine zipper protein ATHB-15 | 381/8e-106 |
| cca-miR169a-1/169a-2/169b | GE593214.1 | AAF79478- F1L3.29 | 121/2e-27 |
| cca-miR172b | GE604670.1 | AAC49773- AP2 domain containing protein RAP2.7 | 124/2e-28 |
| cca-miR393a, b^1^ | JN382008 | ADL70210- transport inhibitor response 1 | 377/3e-105 |
| cca-miR396 | JN382007 | NP_196787- uncharacterized protein | 104/3e-23 |
| cca-miR396d | GE582530.1 | NP_195462- suppressor of RPS4-RLD 1 / tetratricopeptide repeat domain-containing protein | 171/9e-71 |
| cca-miR396f, a^1^ | GE611761.1 | NP_567243- cullin 1 | 131/1e-30 |
| cca-miR397b, d, e^1^ | GE583552.1 | NP_187533- laccase 7 | 351/7e-97 |
| cca-miR398b | GE592589.1 | NP_172360- Superoxide dismutase [Cu-Zn] | 253/2e-67 |
| cca-miR399 | JN382006 | NP_200810- laccase 17 | 153/7e-38 |
| cca-miR403 | GE591170.1 | NP_174413- Argonaute family protein | 154/2e-37 |
| cca-novel-3 | GE590482.1 | NP_974790- zinc finger CCCH domain-containing protein 57 | 196/3e-50 |
| cca-novel-5 | JN382017 | AAF24574- F22C12.18 | 96/5e-20 |
| cca-novel-5 | JN382021 | NP_566227*- ankyrin repeat-containing protein | 86/1e-17 |
| cca-novel-6 | JN382023 | BAE99023- tubulin alpha-2/alpha-4 chain | 126/9e-30 |
| cca-novel-7 | GE600607.1 | NP_199626- scarecrow-like transcription factor PAT1 | 243/2e-64 |
| cca-novel-7 | JN382024 | ABO38767- At2g12550 | 64/2e-17 |
| cca-novel-7/ cca-novel-16-5p | JN382011 | AAM95623- anaphase promoting complex/cyclosome subunit | 66/4e-11 |
| cca-novel-7/ cca-novel-15 | JN382015 | NP_192429- cysteine-rich receptor-like protein kinase 25 | 84/2e-16 |
| cca-novel-8 | GE597991.1 | NP_001190386- cytidine/deoxycytidylate deaminase-like protein | 133/2e-31 |
| cca-novel-8 | GE577335.1 | NP_973859*- zinc finger (C3HC4-type RING finger) family protein | 148/1e-35 |
| cca-novel-8 | JN382014 | NP_195422- PATATIN-like protein 4 | 62/8e-10 |
| cca-novel-8 | JN382019 | NP_563906- photolyase 1 | 91/4e-19 |
| cca-novel-9-3p | GE603783.1 | NP_001032014- beta-amylase | 103/4e-22 |
| cca-novel-9-3p | JN382018 | NP_196846- transaldolase | 101/2e-21 |
| cca-novel-10-5p | GE609807.1 | NP_198716- putative receptor-like protein kinase | 209/3e-54 |
| cca-novel-11 | GE606661.1 | NP_180615- photosystem II reaction center W protein | 104/1e-22 |
| cca-novel-11 | JN382016 | BAF01986.1- Rubisco activase | 67/2e-11 |
| cca-novel-13-3p | GE605842.1 | NP_195958- heavy metal transport/detoxification domain-containing protein | 159/5e-39 |
| cca-novel-14 | GE587322.1 | NP_173513- uncharacterized protein | 133/3e-31 |
| cca-novel-16-3p | JN382022 | NP_974279*- senescence associated protein 20 | 64/3e-11 |
| cca-novel-17 | JN382012 | NP_194958- L-ascorbate peroxidase | 77/2e-14 |
| cca-novel-17 | JN382020 | NP_849433- MAC/Perforin domain-containing protein | 69/1e-12 |
| cca-novel-18 | JN382013 | BAH20208- AT1G11910 (Aspartic proteinasi) | 67/2e-11 |

^1^ Different isomiR targeting the same sequence are reported as: miRNA name followed by comma separated suffix

* Second BLASTx hit according to *E*-value
