# Supplementary material for: The miRNAome of globe artichoke: conserved and novel micro RNAs and target analysis
Source: BMC Genomics. 2012 Jan 24;13:41. doi: 10.1186/1471-2164-13-41 (PMC3285030; doi:10.1186/1471-2164-13-41)
Supplement: Additional file 8 — qPCR primers. Reverse transcription (RT), forward and reverse primers used for artichoke miRNA validation and quantitative real-time PCR analyses. [file 1471-2164-13-41-S8.DOC]

**Additional File 8** Reverse transcription (RT), forward and reverse primers used for artichoke miRNA validation and qPCR analyses, and for actin and elongation factor RT and qPCR.

| Name |  | Primer sequence |
| --- | --- | --- |
| cca-miR156a | RT primer | GTCGTATCCAGTGCAGGGTCCGAGGTATTCGCACTGGATACGACGTGCTC |
|  | Forward primer | GCGGCGGTGACAGAAGAGAGT |
| cca-miR156a* | RT primer | GTCGTATCCAGTGCAGGGTCCGAGGTATTCGCACTGGATACGACGGTGAC |
|  | Forward primer | CGGCGGCTCACTGCTCTATCT |
| cca-miR157a | RT primer | GTCGTATCCAGTGCAGGGTCCGAGGTATTCGCACTGGATACGACGTGCTC |
|  | Forward primer | GCGGCGTTGACAGAAGATAGA |
| cca-miR159a | RT primer | GTCGTATCCAGTGCAGGGTCCGAGGTATTCGCACTGGATACGACTAGAGC |
|  | Forward primer | CGGCGGTTTGGATTGAAGGGA |
| cca-miR160 | RT primer | GTCGTATCCAGTGCAGGGTCCGAGGTATTCGCACTGGATACGACTGGCAT |
|  | Forward primer | CAATGTGCCTGGCTCCCTGT |
| cca-miR164a | RT primer | GTCGTATCCAGTGCAGGGTCCGAGGTATTCGCACTGGATACGACTGCACG |
|  | Forward primer | CGGCGTGGAGAAGCAGGGTA |
| cca-miR166b | RT primer | GTCGTATCCAGTGCAGGGTCCGAGGTATTCGCACTGGATACGACGGGGAA |
|  | Forward primer | ATTAGTCGGACCAGGCTTCA |
| cca-miR167a | RT primer | GTCGTATCCAGTGCAGGGTCCGAGGTATTCGCACTGGATACGACCCAGAT |
|  | Forward primer | TCGCGTGAAGCTGCCAGCAT |
| cca-miR169 | RT primer | GTCGTATCCAGTGCAGGGTCCGAGGTATTCGCACTGGATACGACCCGGCA |
|  | Forward primer | CAATGCAGCCAAGGATGACT |
| cca-miR171a | RT primer | GTCGTATCCAGTGCAGGGTCCGAGGTATTCGCACTGGATACGACGATATT |
|  | Forward primer | CGAAGTGATTGAGCCGTGCC |
| cca-miR172a | RT primer | GTCGTATCCAGTGCAGGGTCCGAGGTATTCGCACTGGATACGACATGCAG |
|  | Forward primer | CGGCGAGAATCTTGATGATG |
| cca-miR319a | RT primer | GTCGTATCCAGTGCAGGGTCCGAGGTATTCGCACTGGATACGACGGGAGC |
|  | Forward primer | CGGCGTTGGACTGAAGGGA |
| cca-miR390 | RT primer | GTCGTATCCAGTGCAGGGTCCGAGGTATTCGCACTGGATACGACGGCGCT |
|  | Forward primer | CAATGAAGCTCAGGAGGGAT |
| cca-miR393a | RT primer | GTCGTATCCAGTGCAGGGTCCGAGGTATTCGCACTGGATACGACGGATCA |
|  | Forward primer | ATTAGTCCAAAGGGATCGCAT |
| cca-miR395 | RT primer | GTCGTATCCAGTGCAGGGTCCGAGGTATTCGCACTGGATACGACGAGTTC |
|  | Forward primer | CAATGCTGAAGTGTTTGGGG |
| cca-miR396a | RT primer | GTCGTATCCAGTGCAGGGTCCGAGGTATTCGCACTGGATACGACAAGTTC |
|  | Forward primer | CGGCGTTCCACAGCTTTCTT |
| cca-miR397a | RT primer | GTCGTATCCAGTGCAGGGTCCGAGGTATTCGCACTGGATACGACCATCAA |
|  | Forward primer | CGAAGTCATTGAGTGCAGCG |
| cca-miR398a | RT primer | GTCGTATCCAGTGCAGGGTCCGAGGTATTCGCACTGGATACGACCAGGGG |
|  | Forward primer | CGAGGTGTGTTCTCAGGTCG |
| cca-miR399a | RT primer | GTCGTATCCAGTGCAGGGTCCGAGGTATTCGCACTGGATACGACCAGGGC |
|  | Forward primer | CGGCGTGCCAAAGGAGATTT |
| cca-miR408a | RT primer | GTCGTATCCAGTGCAGGGTCCGAGGTATTCGCACTGGATACGACAGCCAG |
|  | Forward primer | CGAAGTGCACTGCCTCTTCC |
| cca-miR2910 | RT primer | GTCGTATCCAGTGCAGGGTCCGAGGTATTCGCACTGGATACGACGACAAA |
|  | Forward primer | CAATGTAGTTGGTGGAGCGA |
| cca-novel-1-3p | RT primer | GTCGTATCCAGTGCAGGGTCCGAGGTATTCGCACTGGATACGACCATGCC |
|  | Forward primer | CGGCGCAAGAAGTTGTCTTA |
| cca-novel-10-5p | RT primer | GTCGTATCCAGTGCAGGGTCCGAGGTATTCGCACTGGATACGACGTCATA |
|  | Forward primer | CGGCGGTCTTTATGTCACGATG |
| cca-novel-10-3p | RT primer | GTCGTATCCAGTGCAGGGTCCGAGGTATTCGCACTGGATACGACGCTATT |
|  | Forward primer | CGGCGCATGCATGGTGATATA |
| cca-novel-11 | RT primer | GTCGTATCCAGTGCAGGGTCCGAGGTATTCGCACTGGATACGACCCACTT |
|  | Forward primer | CAGCGGAAGTTTCAAGTGTAAAA |
| cca-novel-8 | RT primer | GTCGTATCCAGTGCAGGGTCCGAGGTATTCGCACTGGATACGACATTCAT |
|  | Forward primer | CAACGATGGACGTGTTATTCATC |
| Universal | Reverse primer | GTGCAGGGTCCGAGGT |
| actin | RT primer | TTCAGTCAGGATCTTCATCAGG |
| actin | Forward primer | TCGCATACAGTGCCAATTTATG |
| elongation factor | RT primer | ATGCTCACGGGTCTGACCATCCTTA |
| elongation factor | Forward primer | TCCCAGGCTGATTGTGCTGTCCTTATTAT |
