# Supplementary material for: The miRNAome of globe artichoke: conserved and novel micro RNAs and target analysis
Source: BMC Genomics. 2012 Jan 24;13:41. doi: 10.1186/1471-2164-13-41 (PMC3285030; doi:10.1186/1471-2164-13-41)
Supplement: Additional file 9 — Target validation primers. Gene specific primers (GSP) used for reverse transcription (GSP1) and PCR amplifications (GSP2 and GSP3) for the validation of miRNA targets. [file 1471-2164-13-41-S9.DOC]

**Additional File 9** Gene specific primers (GSP) used for reverse transcription (GSP1) and PCR amplifications (GSP2 and GSP3) for the validation of miRNA targets

| miRNA Family | Target | Target gene function | 5' RACE primers | |
| --- | --- | --- | --- | --- |
| miR160 | GE577464 | auxin response factor 10 | GSP1 | GCGACCAAAAAGCAGGA |
|  |  |  | GSP2 | TTGAGTTCCGAATGGTAACAGACAG |
|  |  |  | GSP3 | GGTTTGTTCAAGAATGGGTGGATG |
| miR393 | contig_79900 | transport inhibitor response 1 | GSP1 | AGGCATGTCAGACCTT |
|  |  |  | GSP2 | CAACAGGACAGCTATCGAACCTT |
|  |  |  | GSP3 | TCGACATTAAGCATAGGCATTTTC |
| miR397 | GE583552 | laccase 7 | GSP1 | TTGTGCCGTTATTGAAG |
|  |  |  | GSP2 | GCCATGTAATATGATCCTGGTGAC |
|  |  |  | GSP3 | AAAACGATTACGTCGGTTAAGAGC |
| miR398 | GE592589 | superoxide dismutase | GSP1 | GAGATGAGCACAGAAATC |
|  |  |  | GSP2 | TCAGGATCTGCATGAACAACTACA |
|  |  |  | GSP3 | GCCAGAAAGGTTTCCAGTAACAGT |
